# Resuscitation With Early Adrenaline Infusion for Children With Septic Shock: A Randomized Pilot Trial

**DOI:** 10.1097/PCC.0000000000003351

**Published:** 2024-01-19

**Authors:** Amanda Harley, Shane George, Natalie Phillips, Megan King, Debbie Long, Gerben Keijzers, Paula Lister, Sainath Raman, Rinaldo Bellomo, Kristen Gibbons, Luregn J Schlapbach

**Affiliations:** 1 Child Health Research Centre, The University of Queensland, Brisbane, QLD, Australia.; 2 School of Nursing, Midwifery and Social Work, University of Queensland, Brisbane, QLD, Australia.; 3 Department of Emergency Medicine, Gold Coast University Hospital, Southport, QLD, Australia.; 4 Emergency Department Queensland Children`s Hospital, Brisbane, QLD, Australia.; 5 School of Medicine and Menzies Health Institute Queensland, Griffith University, Southport, QLD, Australia.; 6 School of Nursing, Centre of Healthcare Transformation, Queensland University of Technology, Brisbane, QLD, Australia.; 7 Faculty of Health Sciences and Medicine, Bond University, Gold Coast, QLD, Australia.; 8 Children`s Critical Care Unit, Sunshine Coast University Hospital, Birtinya, QLD, Australia.; 9 Paediatric Intensive Care Unit, Queensland Children’s Hospital, Children’s Health Queensland, Brisbane, QLD, Australia.; 10 Intensive Care Research, Austin Hospital and Monash University, Melbourne, VIC, Australia.; 11 Department of Critical Care, University of Melbourne, Melbourne, VIC, Australia.; 12 Australian and New Zealand Research Centre, Monash University, Melbourne, VIC, Australia.; 13 Department of Intensive Care, Royal Melbourne Hospital, Melbourne, VIC, Australia.; 14 Pediatric and Neonatal Intensive Care Unit, and Children`s Research Center, University Children’s Hospital Zurich, Zurich, Switzerland.

**Keywords:** adrenaline, child, emergency department, fluid, inotropes, septic shock

## Abstract

**OBJECTIVES:**

In children with septic shock, guidelines recommend resuscitation with 40–60 mL/kg of fluid boluses, yet there is a lack of evidence to support this practice. We aimed to determine the feasibility of a randomized trial comparing early adrenaline infusion with standard fluid resuscitation in children with septic shock.

**DESIGN:**

Open-label parallel randomized controlled, multicenter pilot study. The primary end point was feasibility; the exploratory clinical endpoint was survival free of organ dysfunction by 28 days.

**SETTING:**

Four pediatric Emergency Departments in Queensland, Australia.

**PATIENTS:**

Children between 28 days and 18 years old with septic shock.

**INTERVENTIONS:**

Patients were assigned 1:1 to receive a continuous adrenaline infusion after 20 mL/kg fluid bolus resuscitation (*n* = 17), or standard care fluid resuscitation defined as delivery of 40 to 60 mL/kg fluid bolus resuscitation prior to inotrope commencement (*n* = 23).

**MEASUREMENTS AND MAIN RESULTS:**

Forty of 58 eligible patients (69%) were consented with a median age of 3.7 years (interquartile range [IQR], 0.9–12.1 yr). The median time from randomization to inotropes was 16 minutes (IQR, 12–26 min) in the intervention group, and 49 minutes (IQR, 29–63 min) in the standard care group. The median amount of fluid delivered during the first 24 hours was 0 mL/kg (IQR, 0–10.0 mL/kg) in the intervention group, and 20.0 mL/kg (14.6–28.6 mL/kg) in the standard group (difference, –20.0; 95% CI, –28.0 to –12.0). The number of days alive and free of organ dysfunction did not differ between the intervention and standard care groups, with a median of 27 days (IQR, 26–27 d) versus 26 days (IQR, 25–27 d). There were no adverse events reported associated with the intervention.

**CONCLUSIONS:**

In children with septic shock, a protocol comparing early administration of adrenaline versus standard care achieved separation between the study arms in relation to inotrope and fluid bolus use.

**RESEARCH IN CONTEXT**

- While liberal fluid-based resuscitation has represented a hallmark of pediatric septic shock treatment, more recently, accumulating observational evidence suggests that excess administration of fluid can result in harm.
- To date, there is a lack of clinically directive data on whether starting inotropes early to reduce the administration of resuscitation fluid is feasible, safe, and whether it may be superior compared to standard resuscitation in children with sepsis.
- It is unknown if a protocol comparing early adrenaline infusion (started after 20 mL/kg fluid bolus) versus standard care (40–60 mL/kg fluid bolus followed by inotrope infusion) is feasible in children with septic shock.

**WHAT THIS STUDY MEANS**

- In this randomized trial including 40 children with septic shock, a protocol comparing early adrenaline infusion versus standard care was feasible, but recruitment rates were low.
- Inotropes were commenced at a median of 16 minutes after randomization in the intervention group compared with 49 minutes in the standard group, and the median amount of IV fluid bolus volume received in the first 24 hours was 0 mL/kg in the intervention group, and 20.0 mg/kg in the standard group.
- Organ dysfunction-free survival, rates of PICU admission, PICU length of stay, and safety outcomes were similar between the study arms.

Septic shock is characterized by profound circulatory, cellular, and metabolic dysfunction as a result of the body’s dysregulated response to infection (1). Mortality in children with septic shock ranges from 17% to 32% (2, 3). The Surviving Sepsis Campaign (SSC) recommends an initial treatment bundle consisting of broad-spectrum antibiotics, blood culture sampling, and 40–60 mL/kg of IV fluid, which must be initiated within 1 hour of recognizing septic shock (4). The administration of 40–60 mL/kg of IV fluid was classified as a weak recommendation as the optimal amount of fluid resuscitation for children remains unknown (5). Referring to the Fluid Expansion as Supportive Therapy (FEAST) trial (6), the SSC guidelines recommend against initial fluid-based resuscitation in settings where no intensive care can be provided.

While liberal fluid-based resuscitation has represented a hallmark of pediatric septic shock treatment, accumulating observational evidence suggests that excess administration of fluid can result in harm (7). An analysis of 3,969 children reported that a higher fluid bolus administration rate was associated with increased adjusted odds of death, and of mechanical respiratory support (8). The rationale in delivering IV fluid in septic shock is founded upon the assumption that fluid boluses can restore hemodynamic stability and improve tissue oxygen delivery (9). Yet, septic children may not be hypovolemic, and they often manifest impaired cardiac function combined with variable degrees of vasoplegia (10). To date, there is a lack of clinically directive data on whether starting inotropes early to reduce the administration of resuscitation fluid is feasible, safe, and whether it may be superior compared to standard resuscitation in children with sepsis.

We performed the Early Resuscitation in Paediatric Sepsis Using Inotropes—A Pilot Randomised Controlled Pilot Study in the Emergency Department (RESPOND ED) study to investigate the feasibility of early adrenaline infusion (after 20 mL/kg IV fluids) compared to standard sepsis resuscitation (delivery of 40–60 mL/kg IV fluid before starting inotropes) in children with sepsis.

## METHODS

### Study Design and Oversight

The RESPOND ED study was a pilot multicenter, open label, nonblinded, pragmatic randomized controlled trial (RCT) conducted in four specialized pediatric Emergency Departments (EDs) in Queensland, Australia between July 2019 and August 2021. The Child Health Research Center (University of Queensland, Brisbane, QLD, Australia) managed the trial (**Supplementary Material S1**, http://links.lww.com/PCC/C422). The trial was registered in the Australian New Zealand Clinical Trials Registry before the start of enrollment (ACTRN12619000828123). The trial protocol including the statistical analysis plan have been published before completion of enrollment (11). Approval from the Human Research Ethics Committee (HREC) (HREC/18/QCHQ/49168, **Supplementary Material S2**, http://links.lww.com/PCC/C422) was obtained for all sites before enrollment commenced. Written informed consent or written consent to continue was obtained for all participants. The Data and Safety Monitoring Board controlled trial conduct and safety (**Supplementary Material S3**, http://links.lww.com/PCC/C422). Co-enrollment in a pilot trial evaluating feasibility of hydrocortisone, vitamin C, and thiamine in the PICU was allowed (ACTRN12619000829112 [12]) (13).

### Patients and Settings

Screening was performed 24/7 at participating sites by study coordinators. Participants included were children between 28 days and 18 years old treated for septic shock. Children were eligible if the treating clinician decided to continue treating for septic shock after the delivery of a minimum of 20 mL/kg fluid bolus in the previous 4 hours (or a total of 1,000 mL in patients ≥ 50 kg). Children were excluded if fluid bolus treatment had exceeded 40 mL/kg in the 4 hours prior to enrollment. Full inclusion and exclusion criteria are shown in **Supplementary Material S4** (http://links.lww.com/PCC/C422).

### Randomization

Eligible participants were randomized to a 1:1 ratio to the intervention (early adrenaline infusion) and standard care group. A permuted block randomization method with block sizes of two, four, and six with site stratification was used to allocate patients to a study group, and opaque sealed envelopes stored in a secure research cupboard located within the ED were used for randomization. No blinding was performed due to the logistic difficulties in blinding IV fluid bolus versus adrenaline infusion.

### Interventions

Patients in the intervention arm were started on an infusion of IV adrenaline immediately after randomization, that is, after the initial fluid bolus of 20 mL/kg (1,000 mL fluid bolus in patients ≥ 50 kg). Adrenaline was initiated at 0.05 to 0.1 (upto 0.3) microgram/kg/min as per institutional guidelines and was given through a peripheral IV, intraosseous, or central venous access line as available. Instructions were given to deliver adrenaline for at least 60 minutes at a dose titrated to age-based physiological targets in patients who were considered stable by the treating physician before weaning the drug. Patients who were successfully weaned off adrenaline within less than 4 hours were allowed to be admitted to the ward based on judgment by the treating physician; the protocol recommended a PICU review within 4–6 hours after transfer from ED. In patients who deteriorated despite adrenaline infusion, increase of the dose rate and admission to PICU as per local practice was recommended. Other management such as choice of fluid type, titration of additional fluid boluses and inotropes, antibiotics, and respiratory support were not prescribed.

### Study Endpoints

RESPOND ED assessed feasibility outcomes, and exploratory clinical primary and secondary outcomes (14). Feasibility outcomes were defined as recruitment rates (targeting recruitment of ≥ 65% of eligible patients), time to initiation of inotropes (targeting 20 min earlier inotrope commencement in the early inotrope arm), fluid delivery during the first 24 hours (targeting 10 mL/kg lower fluid bolus administration during the first 24 hr in the early inotrope arm), and protocol violations. The exploratory primary clinical outcome was survival free of organ dysfunction censored at 28 days. Organ dysfunction was defined as pediatric Sequential Organ Failure Assessment score greater than 0 (15, 16). Patients dying within 28 days of presentation were considered as 0 days. Secondary outcomes included survival free of inotrope support and free of multiple organ dysfunction at 7 days, 28-day mortality, survival free of PICU, as well as hospital and PICU length of stay. In all patients, Pediatric Overall Performance Category (POPC) (17) and Functional Status Score (FSS) (18) were recorded at enrollment (baseline) as well as 28 days post-randomization. We recorded the amount of IV bolus fluid (mL) received by 1, 4, 12, and 24 hours; the proportion of patients with a lactate level less than 2 mmol/L at 6, 12, and 24 hours; time to reversal of tachycardia; and time to shock reversal defined as cessation of inotropes for at least 4 hours.

### Data Collection

Data were collected via a study booklet and was entered into a secure Research Electronic Data Capture database, hosted by The University of Queensland. All randomized patients were followed up for 28 days post-randomization. Organ dysfunction scores and organ support modes were captured daily for patients in PICU, and, for patients discharged from ED to the ward not requiring PICU, for the day beyond the day of randomization. We performed independent primary source data verification in all enrolled patients for eligibility, consent, study interventions, organ support PICU admission and discharge dates, and survival status. In a random selection of 10% of patients, additional study fields were monitored (**Supplemental Materials S5**, http://links.lww.com/PCC/C422).

### Statistical Analysis

This study was designed as a feasibility RCT not powered for clinical outcomes. We estimated a priori a recruitment rate of one patient per month at the lead site, enabling completion of the trial in 2 years. The statistical analysis plan was published before completion of enrollment with the full Stata analysis code publicly available through GitHub (San Francisco, CA) (14). Analyses were performed on the intention-to-treat population including all eligible and enrolled children except those who were randomized but where parents did not provide consent-to-continue. Descriptive statistics were utilized to report on baseline clinical characteristics. The feasibility and clinical outcome measures were compared with the estimate of the difference and corresponding 95% CIs. For continuous outcomes quantile regression was used to generate the CIs. A test of two proportions was used for the binary outcomes. All analyses were performed with Stata/SE Version 17.0 (StataCorp, LLC, College Station, TX).

## RESULTS

### Patients and Recruitment Rates

**Figure 1.**
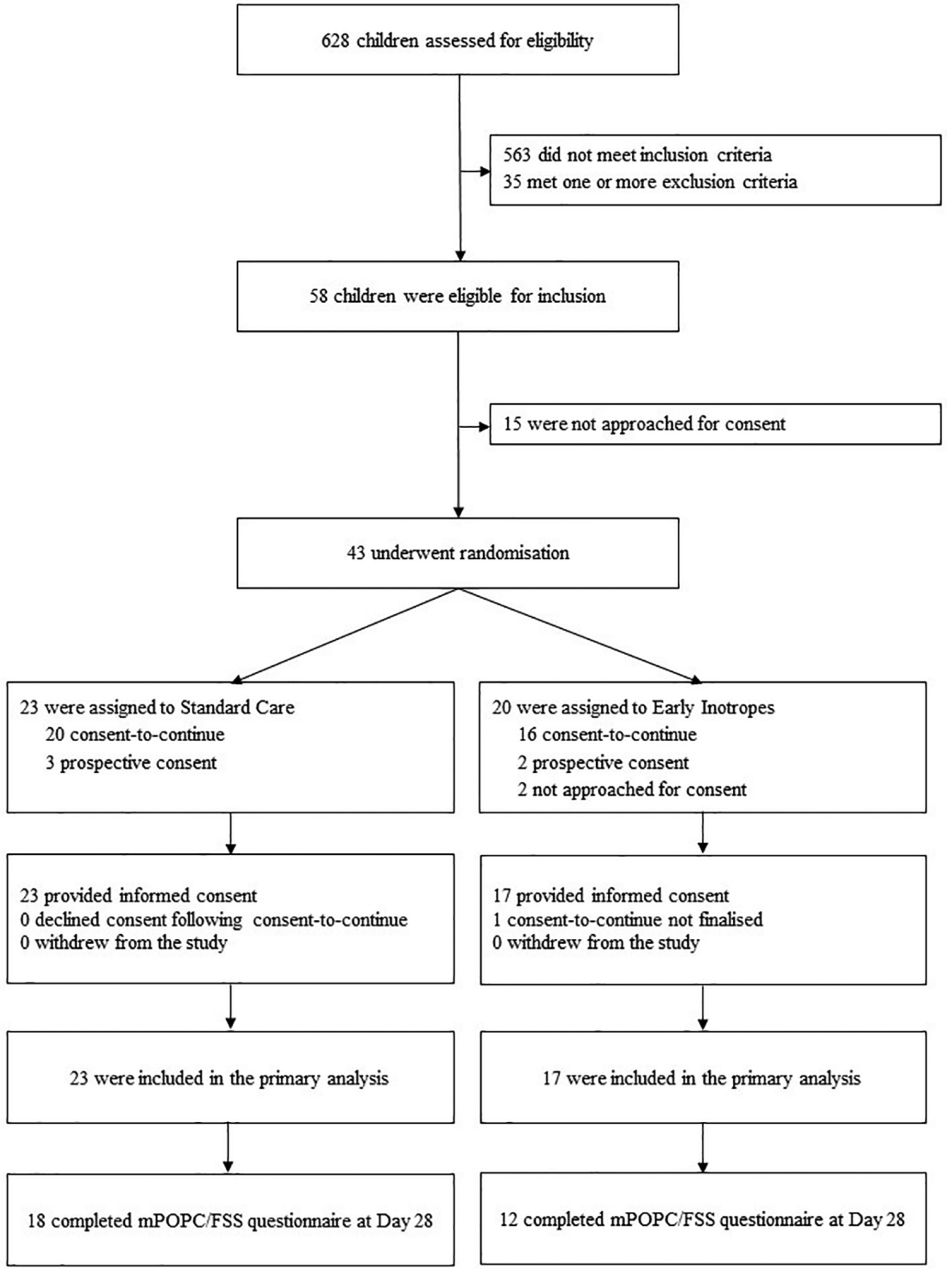
Consolidated Standards of Reporting Trials participant flow diagram for the Randomised Controlled Pilot Study in the Emergency Department trial. FSS = Functional Status Score, mPOPC = modified Pediatric Overall Performance Category.

From June 1, 2019, to August 8, 2021, we screened 628 children. Out of 58 eligible patients, 17 (29%) were not approached for consent and 1 (2%) was not suitable for consent due to child custody issues. Consent-to-continue was employed in 36 cases (84%). No parents declined consent leaving a modified intention-to-treat population of 40 enrolled patients. Average monthly recruitment rate was 1.5 patients across all sites and 1.2 patients at the lead site. The median age was 3.7 years (interquartile range [IQR], 0.9–12.1 yr) (**Table 1**). Seventeen (43%) were enrolled into the early adrenaline intervention, and 23 to standard care (**Fig. 1**; and **Fig. S1**, http://links.lww.com/PCC/C422). The median total amount of IV fluid bolus volume received at time of randomization was 19.4 mL/kg (IQR, 18.6–20.3 mL/kg) in the intervention group, and 19.9 mL/kg (19.2–20.6 mL/kg) in the standard group.

**TABLE 1.** Baseline Characteristics of Infants Enrolled in the Randomised Controlled Pilot Study in the Emergency Department Trial

Characteristic	Variable	Standard Care (*n* = 23)	Early Inotropes (*n* = 17
Demographics	Age at randomization (yr)	6.5 (0.8–14.5)	2.1 (0.9–6.9)
	Weight (kg)	18.6 (9.6–48.5)	12.9 (10.8–25.8)
	Female sex	11 (48%)	8 (47%)
Ethnicity	Caucasian	5 (22%)	4 (24%)
	Aboriginal/Torres Strait Islander	2 (9%)	1 (6%)
	Asian	3 (13%)	2 (12%)
	Maori/Pacific Islander	0 (0%)	1 (6%)
	Mixed/other	1 (4%)	2 (12%)
	Unknown	12 (52%)	7 (41%)
Comorbidities	Chronic disease	8 (35%)	3 (18%)
	Congenital malformation	1 (4%)	0 (0%)
	Congenital heart defect	2 (9%)	1 (6%)
	Oncologic disease	3 (13%)	0 (0%)
	Cerebral palsy/severe encephalopathy	2 (9%)	0 (0%)
	Syndrome/genetic disorder	3 (13%)	1 (6%)
Observations at baseline	Heart rate (*n* = 38)	128 (115–172)	169 (147–183)
	Respiratory rate (*n* = 36)	36 (26–54)	35 (25–53)
	Systolic blood pressure (*n* = 37)	92 (86–105)	94 (81–99)
	Temperature(*n* = 31)	37.9 (37.4–39.1)	38.8 (37.3–39.1)
	Oxygen saturation (*n* = 38)	98 (97–100)	98 (97–100)
	High-flow nasal cannula or noninvasive respiratory support	2 (9%)	0 (0%)
	Invasive respiratory support	0 (0%)	1 (6%)
Alert, Voice, Pain, Unresponsive score (*n* = 35)	Alert	16 (89%)	10 (59%)
	Voice	2 (11%)	3 (18%)
	Pain	0 (0%)	2 (12%)
	Unresponsive	0 (0%)	2 (12%)
Laboratory investigations	pH (*n* = 35)	7.39 (7.35–7.42)	7.35 (7.26–7.38)
	Base excess (mmol/L) (*n* = 23)	–3.1 (–6.3 to –1.8)	–5.4 (–7.6 to –1.6)
	Pco_2_ (mm Hg) (*n* = 35)	36.0 (32.0–44.0)	40.0 (36.5–50.0)
	Lactate (mmol/L) (*n* = 33)	2.0 (1.1–3.2)	2.3 (2.0–4.0)
	Creatinine (µmol/L) (*n* = 33)	43 (29–78)	38 (31–59)
	International normalized ratio (*n* = 9)	1.3 (1.2–1.4)	1.2 (1.2–1.3)
	Fibrinogen (g/L) (*n* = 9)	4.7 (2.7–5.4)	3.3 (2.8–3.8)
	Platelets (×10^3^/µL) (*n* = 32)	220 (158–350)	285 (249–389)
	White cell count (×10^3^/µL) (*n* = 33)	10.2 (5.1–18.7)	9.4 (6.9–14.1)
	C-reactive protein (mg/L) (*n* = 29)	79 (45–218)	10 (4–136)
Organ dysfunction score	Pediatric Sequential Organ Failure Assessment	2 (1–3)	4 (2–6)
Treatment	Total amount of fluid boluses received within the past 4 hr (mL/kg)	19.9 (19.2–20.6)	19.4 (18.6–20.3)

Values are expressed as *n* (%) and median (interquartile range).

### Interventions

Overall, the median time from screening to randomization was 15 minutes (IQR, 0–36 min). Thirteen patients (76%) in the intervention group, and 9 (39%) and the standard care group were started on IV inotrope infusions within the first hour of enrollment, with a median time from randomization to inotropes of 16 minutes (IQR, 12–26 min) versus 49 minutes (IQR, 29–63 min), respectively (**Table 2**). In four patients (25%) allocated to the early adrenaline arm, clinicians decided not to start an inotrope. The median amount of IV fluid bolus volume received in the first 24 hours after randomization was 0 mL/kg (IQR, 0–10.0 mL/kg) in the intervention group, and 20.0 mL/kg (IQR, 14.6–28.6 mL/kg) in the standard group. The median duration of inotropes was 4.4 hours (IQR, 1.6–10.2 hr) in the intervention group and 5.0 hours (IQR, 0–24.1 hr) in the standard care group.

**TABLE 2.** Feasibility Outcomes per Intention-to-Treat Analysis

Outcome	Standard Care (*n* = 23)	Early Inotropes (*n* = 17)	Estimate of Difference (95% CI)
Time from screening to randomization (min)	19 (0–73)	15 (0–32)	–4 (–47 to 39)
Time from randomization to adrenaline infusion commencement (min)	42 (20–63) (*n* = 11)	17 (12–27) (*n* = 13)	–25 (–71 to 21)
Time from randomization to any inotrope infusion commencement (min)	49 (29–63) (*n* = 13)	16 (12–26) (*n* = 13)	–33 (–76 to 10)
Inotrope infusion received within first hour of randomization	9 (39%)	13 (76%)	37% (9–66%)
Inotrope infusion received within 24 hr of randomization	13 (57%)	13 (76%)	20% (–9% to 49%)
Amount of fluid received during the first 24 hr (mL/kg)	20.0 (14.6–28.6)	0 (0–10.0)	–20.0 (–28.0 to –12.0)
IV fluid volume received during the first 1 hr (mL/kg)	11.4 (10.0–18.7)	0 (0–9.4)	–11.4 (–16.9 to –5.8)
IV fluid volume received between > 1 and 4 hr (mL/kg)	0 (0–10.0)	0 (0–0)	0 (–5.5 to 5.5)
IV fluid volume received between > 4 and 12 hr (mL/kg)	0 (0–5.2)	0 (0–0)	0 (–3.3 to 3.3)
IV fluid volume received between > 12 and 24 hr (mL/kg)	0 (0–0)	0 (0–0)	Not applicable
Later enrollment in Resuscitation in Paediatric Sepsis Randomized Controlled Pilot Platform Study in the Paediatric Intensive Care Unit (RESPOND PICU)	6 (26%)	2 (12%)	–14 (–38% to 9%)

Values are expressed as *n* (%) and median (interquartile range).

### Exploratory Clinical Endpoints

**Figure 2.**
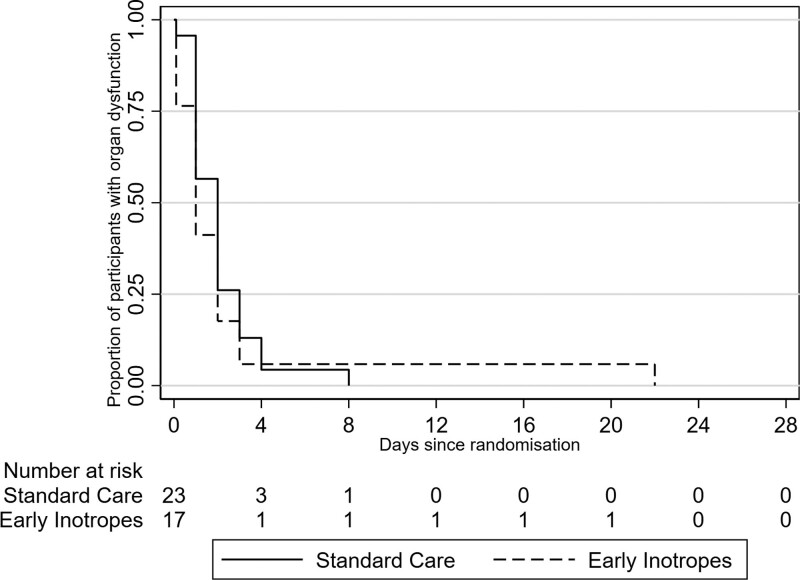
Cumulative incidence functions for survival free of organ dysfunction (accounting for mortality) for the study cohort.

In children allocated to early adrenaline infusion, the median number of organ dysfunction-free days at 28 days was 27 days (IQR, 26–27 d) compared with 26 days (IQR, 25–27 d) in the standard care group (unadjusted estimate of absolute difference, 1 d; 95% CI, –0.1 to 2.1 d) (**Table 3** and **Fig. 2**). Twelve patients (71%) in the intervention group, and 20 (87%) in the standard care group were admitted to PICU, with a median PICU length of stay of 2.0 days (IQR, 0.8–2.6 d) in the intervention group and 2.2 days (IQR, 1.0–3.2 d) in the standard care group (estimate of difference, –0.4; 95% CI, –1.9 to 1.0). The median hospital length of stay was shorter in the intervention group (–3.2 d; 95% CI, –6.1 to –0.3 d). No patients died within 28 days after randomization. One (6%) and five (22%) patients in the intervention and the standard care group, respectively, received hydrocortisone after randomization.

**TABLE 3.** Distribution of Candidate Clinical Outcomes for a Definitive Trial per Intention-to-Treat Analysis

Outcome	Standard Care (*n* = 23)	Early Inotropes (*n* = 17)	Estimate of Difference (95% CI)
Primary clinical outcome			
Survival free of organ dysfunction^a^ censored at 28 d	26.0 (25.0–27.0)	27.0 (26.0–27.0)	1 (–0.1 to 2.1)
Secondary clinical outcomes			
Survival free of inotrope support at 7 d	6.8 (6.0–7.0)	6.8 (6.6–6.9)	–0.02 (–0.6 to 0.5)
Survival free of multiple organ dysfunction^b^ at 7 d	5.0 (5.0–6.0)	6.0 (5.0–6.0)	1 (–0.1 to 2.1)
28-d mortality	0 (0%)	0 (0%)	Not applicable
Survival free of PICU censored at 28 d	26.4 (24.9–27.3)	27.2 (25.8–28.0)	0.8 (–0.6 to 2.3)
Length of stay in PICU	2.2 (1.0–3.2) (*n* = 20)	2.0 (0.8–2.5) (*n* = 12)	–0.4 (–1.9 to 1.0)
Length of stay in hospital	6.8 (3.9–9.3)	3.6 (2.7–5.5)	–3.2 (–6.1 to –0.3)
Modified POPC at 28 d	2 (1–3) (*n* = 16)	1 (1–2) (*n* = 13)	–1 (–1.9 to –0.06)
Change in modified POPC from baseline	0 (0–0.5) (*n* = 16)	0 (0–0) (*n* = 13)	0 (–0.6 to 0.6)
FSS at 28 d	6 (6–7) (*n* = 16)	6 (6–8) (*n* = 11)	0 (–1.2 to 1.2)
Change in FSS from baseline	0 (0–0) (*n* = 16)	0 (0–0) (*n* = 11)	0 (–0.9 to 0.9)
Proxy measures of intervention efficacy			
Proportion with lactate < 2 mmol/L by 6 hr post-randomization	9 (39%)	5 (29%)	–10% (–40% to 20%)
Proportion with lactate < 2 mmol/L by 12 hr post-randomization	13 (57%)	7 (41%)	–15% (–46% to 16%)
Proportion with lactate < 2 mmol/L by 24 hr post-randomization	16 (70%)	10 (59%)	–11% (–41% to 20%)
Time to reversal of tachycardia censored at 24 hr (hr)	1.3 (0–3.7)	2.0 (0.7–4.0)	0.7 (–1.5 to 2.8)
Time to shock reversal censored at 28 d (hr)	5.1 (0–25.2)	4.8 (2.6–10.3)	–0.2 (–14.3 to 13.9)

FSS = Functional Status Score, POPC = Pediatric Overall Performance Category.

a As measured by pediatric Sequential Organ Failure Assessment (pSOFA) score.

b Multiple organ dysfunction is defined as > 1 organ with a pSOFA subscore of > 0.

Values are expressed as *n* (%) and median (interquartile range).

In children in the intervention group, shock reversal defined as cessation of inotropes for at least 4 hours occurred at a median of 4.8 hours (IQR, 2.6–10.3 hr), as compared with 5.1 hours (IQR, 0–25.2 hr) in the standard group (estimate of difference, –0.2; 95% CI, –14.3 to 13.9; Table 3). We explored the dynamics of lactate levels, reversal of tachycardia, shock index, fluid bolus volume (in mL/kg), and Vasopressor-Inotrope Score (19) during the first 24 hours after randomization between the intervention group and the standard group (**Fig. S2**, http://links.lww.com/PCC/C422). At 28 days, the median POPC and FSS were 1 (IQR, 1–2) and 6 (IQR, 6–8) in the intervention group compared with 2 (IQR, 1–3) and 6 (6–7) in the standard group (Table 3; and **Fig. S3**, http://links.lww.com/PCC/C422).

### Protocol Violations and Adverse Events

We recorded seven protocol violations in six patients in the intervention group, and three violations in two patients in the standard group, respectively (**Table S1**, http://links.lww.com/PCC/C422). The most common protocol violation was attributed to clinicians not administering inotropes in the intervention arm. We recorded no adverse events in either group. Specifically, no limb ischemia or extravasation injury occurred.

## DISCUSSION

In this multicenter open label randomized pilot trial of children presenting with early septic shock to ED, a sepsis resuscitation algorithm incorporating early initiation of adrenaline infusion after 20 mL/kg fluid bolus was associated with significantly more and earlier inotrope use, and less fluid volume load during the first 24 hours after randomization as compared with standard SSC resuscitation with 40–60 mL/kg fluid. The intervention did not lead to more or longer PICU admissions. The consent rate was 100% among families approached for consent, and consent-to-continue was used in over 80% of cases given the time constraints associated with the intervention. However, only 69% of eligible patients were enrolled, and the recruitment rate was 1.2 per month at the largest study site.

Founded on concerns about high mortality associated with delayed sepsis resuscitation (20), previous pediatric sepsis guidelines have emphasized the importance of rapid delivery of 40–60 mL/kg fluid bolus during initial resuscitation (21–25). The FEAST study (6) demonstrated a 45% (95% CI, 13–86%) relative mortality risk increase associated with any 20 to 40 mL/kg bolus compared with no bolus. Contrary to septic adults, where the recent Conservative versus Liberal Approach to Fluid Therapy of Septic Shock in Intensive Care (CLASSIC) and Crystalloid Liberal or Vasopressors Early Resuscitation in Sepsis (CLOVERS) trials showed that a restrictive compared to a liberal fluid resuscitation regimen had similar outcomes (26, 27), no comparable data are available for children, and controversy surrounding optimal fluid volume persists. Although the 2020 pediatric SSC recommendations emphasize that in settings where no intensive care support is available, fluid boluses should be avoided in the absence of hypotension (28), the guidelines otherwise still recommend 40–60 mL/kg fluids.

In septic children, only few randomized studies have investigated fluid sparing strategies. The Canadian SQUEEZE trial (NCT03080038) is enrolling children with septic shock who received 40 mL/kg (29), compared with 20 mL/kg which was used in our trial. The U.K. Fluids in Shock pilot trial randomized 73 septic children with hypotension and/or prolonged capillary refill after 20 mL/kg to a restricted (10 mL/kg) versus a standard (20 mL/kg) fluid bolus. The intervention was associated with a –11.2 mL/kg (95% CI, –16.6 to –5.8 mL/kg) reduction of fluid volume administered during the initial hours. Inotrope use was not mandated and only 8% of patients received inotropes compared with 65% in our study. The acuity in both studies was low with zero mortality. The severity of illness observed in our trial thus stands in stark contrast to reports from low- and middle-income settings. An Indian RCT comparing a modified early goal-directed strategy (40 mL/kg fluid delivered over 15 min followed by dopamine) versus standard care (60 mL/kg fluid delivered over 60 min followed by dopamine) reported a mortality of 17.6% (30). Notably, this study observed indices of harmful fluid overload such as hepatomegaly, and a trend toward longer ventilation duration associated with faster fluid administration. A recent Indian RCT compared fluid bolus delivery over 10–20 minutes versus 5–10 minutes in 96 children and faster bolus delivery led to worse oxygenation markers (31). A nonrandomized single-center study in India investigated a protocol using early noradrenaline in children with septic shock (32). Compared with 41 children treated as per American College of Critical Care Medicine guidelines, the 27 patients receiving early noradrenaline received significantly lower amounts of IV fluids and required significantly shorter ventilatory and PICU support. Collectively, these studies underpin the necessity to study fluid-restrictive resuscitation strategies for children with septic shock (20, 33).

The findings from our trial indicate that a protocol using early inotropes in children with septic shock enables faster initiation of inotropes and sparing of fluids during sepsis resuscitation. The intervention appeared to be safe with no extravasation injuries observed, and it was not associated with longer PICU or hospital stay. The trial population was defined by a pragmatic point of enrollment, reflecting a clinician’s decision to treat a child for septic shock with IV antibiotics and fluids rather than prescribing specific blood pressure, perfusion, or systemic inflammatory response syndrome criteria (34–36). However, based on our findings it appears implausible to perform such a trial powered for mortality in high-income settings due to the low severity. A trial powered to detect a difference of at least 12 hours in ICU-free survival censored at 7 days, assuming type I error of 0.05, power of 90%, inflation of 15% for non-normally distributed outcome (sd 1.9 d), would require 358 patients per arm (716 patients total). While the platform design allowed to optimize study education, screening, recruitment, and data collection processes for RESPOND ED with the concomitantly conducted RESPOND PICU trial, recruitment of RESPOND PICU remained substantially higher and included a much sicker cohort (13). Overall, our findings thus provide rationale to test a similar protocol in a less resourced environment. Specifying entry criteria for shock, such as hypotension in presence of increased lactate may permit to select a population of higher severity, and further simplification of exclusion criteria will increase generalizability. Furthermore, consideration to allow use of either adrenaline or noradrenaline in the intervention arm may be warranted. We defined reversal of shock to assess duration of inotropes as a balancing measure, but this construct may be unnecessary. Finally, a primary outcome of ICU-free survival with weighting of death as a worst outcome would reduce the workload of data collection compared with organ dysfunction-free survival. All together these limitations provide rationale to perform an alternative pilot study in a more resource limited setting given different logistical challenges and epidemiology.

Limitations of this pilot RCT include, first, that the intervention was not blinded for logistic and safety reasons. Second, the study protocol mandated use of adrenaline in the intervention arm (10), although the SSC recommends to use either adrenaline or noradrenaline (4, 37). We opted for adrenaline because ED staff at the study sites were more familiar with adrenaline, and as we did not want to guide choice of inotrope by echocardiography, given the SSC recommendation against categorization into warm vs cold shock based on clinical bedside markers alone (4). Third, we did not prescribe the type of resuscitation fluid used, despite increasing evidence about superiority of balanced crystalloids (38). Fourth, the study was performed in pediatric EDs in Southeast Queensland which had participated previously in a statewide sepsis quality improvement initiative (39, 40). Finally, the overall acuity in the study cohort was low, and most patients did well even without further fluids or inotropes, hindering the ability to comment on severity outcomes (21).

In conclusion, our findings demonstrate that a fluid-sparing algorithm for children presenting to the ED with septic shock using early adrenaline is feasible and provide rationale for performing such a trial in children.

## ACKNOWLEDGMENTS

We would like to express our gratitude toward the parents and children participating in this trial as well as to thank the clinical and research teams in the study sites for their help in study conduct.

## Supplementary Material


